# Dynamic tail amine interactions drive isoform selectivity for potent human neuronal nitric oxide synthase inhibitors

**DOI:** 10.1007/s00044-026-03552-3

**Published:** 2026-04-10

**Authors:** Amardeep Awasthi, Koon Mook Kang, Richard B. Silverman

**Affiliations:** 1 Department of Chemistry, Chemistry of Life Processes Institute, Center for Developmental Therapeutics, Northwestern University, 2145 Sheridan Road, Evanston, Illinois 60208-3113 US; 2 Department of Molecular Biosciences, Northwestern University, Evanston, Illinois 60208 US; 3 Department of Pharmacology, Feinberg School of Medicine, Northwestern University, Chicago, Illinois 60611 US

**Keywords:** neuronal nitric oxide synthase, isoform selectivity, cation–π interaction, structure-based drug design, molecular dynamics, CNS permeability

## Abstract

Achieving isoform-selective inhibition of neuronal nitric oxide synthase (nNOS) remains a significant challenge due to the high structural similarity with other NOS isoforms. Here, we report the design, synthesis, and characterization of novel nNOS inhibitors **3** and **4**, incorporating dimethylamino-substituted tail groups to exploit hnNOS-specific peripheral pocket interactions. Both compounds retained sub-20 nM potency against human nNOS with enhanced selectivity over endothelial (hn/he > 1500-fold) and inducible (hn/hi > 229-fold) isoforms. Molecular dynamics simulations and MM-GBSA calculations suggested that hnNOS selectivity arises from a dynamically formed cation-π interaction between the terminal amino group and W311(B), which is precluded in heNOS due to the steric hindrance from F105. PAMPA-BBB assays inḍdicated moderate blood-brain barrier permeability, supporting CNS applications. These findings highlight peripheral pocket interactions as key drivers of isoform selectivity and guide future nNOS inhibitor optimization for neurodegenerative diseases and melanoma.

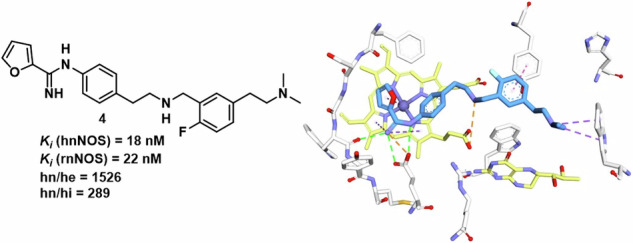

## Introduction

Nitric oxide synthases (NOSs) are a family of heme-containing enzymes responsible to produce nitric oxide (NO), a critical signaling molecule involved in neurotransmission, vascular regulation, and immune response [1, 2]. NOSs are homodimeric enzymes, in which each monomer contains a C-terminal reductase domain and an N-terminal oxygenase domain. The reductase domain consists of reduced nicotinamide adenine dinucleotide phosphate (NADPH), flavin mononucleotide (FMN) and flavin adenine dinucleotide (FAD), while the oxygenase domain accommodates substrate L-arginine, cofactor tetrahydrobiopterin (BH_4_), catalytic heme, and non-catalytic zinc (Zn^2+^) [1, 3, 4]. These domains are linked through a calmodulin-binding region, which modulates NOSs activity proportional to the calcium levels [1, 3]. The reductase domain facilitates electron transfer from NADPH, through FAD and FMN, to the iron center of heme in the catalytic site of the oxygenase domain. This triggers the conversion of L-Arg to L-citrulline in the presence of molecular oxygen while producing nitric oxide in the process [1–3].

**Fig. 1:**
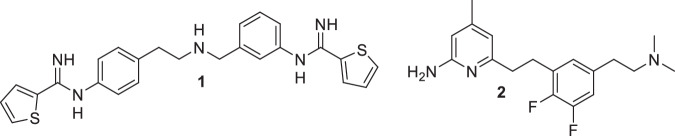
Chemical Structures of Previous Leads **1** and **2**

Among the three major mammalian isoforms, neuronal (nNOS), endothelial (eNOS), and inducible (iNOS) NOSs, selective inhibition of nNOS has attracted significant interest for the treatment of neurological disorders and melanoma [5–9]. However, achieving high isoform selectivity remains challenging due to the high degree of similarity in the binding pocket of these isoforms [10–13]. [14, 15] Previously, the Silverman group has developed several classes of potent nNOS inhibitors, including aminopyridine-based and thiophene-based scaffolds with promising preclinical potential [13]. Particularly, **1** and **2**, showed excellent potency toward human nNOS (*K*_i_ 20 nM and 19 nM, respectively), with 337 and 1075-fold selectivity over human eNOS, respectively **(**Fig. 1**)** [16, 17].

In this work, we employed a hybrid rational design strategy by analyzing the binding modes of **1** and **2** to design new lead molecules **3** and **4** with improved potency and selectivity of nNOS inhibitors. Through molecular dynamics simulations, we investigated the binding behavior of **4** in both hnNOS and heNOS. By integrating structural dynamics with molecular mechanics/generalized born surface area (MM-GBSA) free energy calculations, we proposed that hnNOS selectivity arises from a dynamically formed cation-π interaction by the amine tail unique to the hnNOS peripheral pocket. These findings provide binding insight into NOS isoform selectivity that can help in the rational design of the next generation of hnNOS selective inhibitors.

## Results & discussion

### Design rationale

**Fig. 2:**
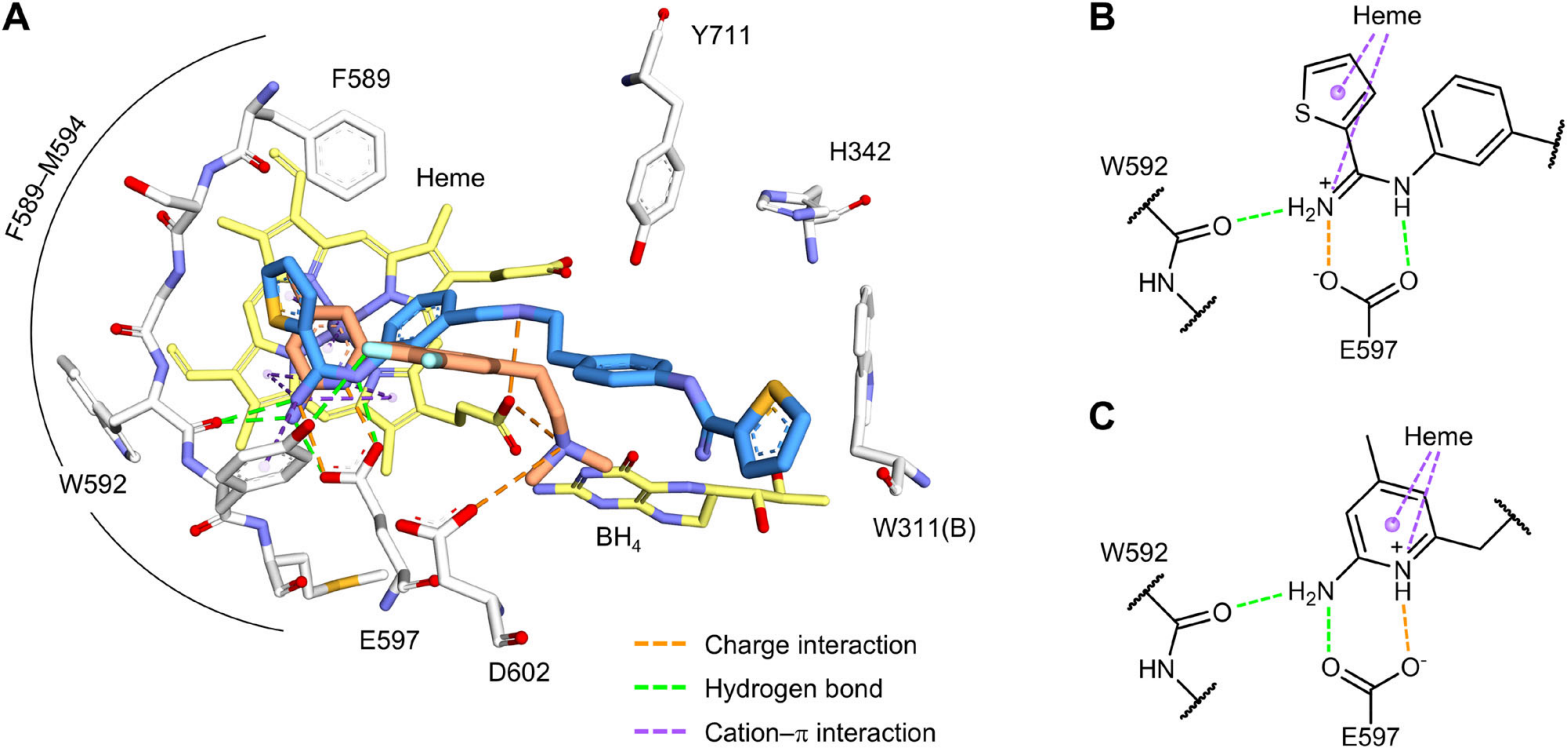
(**A**) Active site view of hnNOS with ligands **1** (PDB 9MWA) and **2** (PDB 8FGG) superimposed. White, yellow, blue, and orange sticks represent the hnNOS active site residues, cofactor atoms, **1**, and **2**, respectively. (**B,** **C**) Interaction diagrams for the head-groups of **1** (**B**) and **2** (**C**).

Previously, the Silverman group reported crystal structures of **1** and **2** bound to hnNOS [16, 17]. Both lead molecules shared a similar head-group binding mode, forming a salt-bridge with E597, accounting for their excellent nanomolar potency **(**Fig. 2**)**. However, substantial differences emerged in their linker and tail-group orientations within the binding pocket.

The aminopyridine based inhibitor **2** positioned its tail group in an upward binding mode within the hnNOS active site. The difluorobenzene moiety was located near Y567, enabling hydrogen bonding interactions, and the terminal amino group engaged with the hnNOS-specific residue Asp602 and heme propionates. In contrast, the thiophene-based inhibitor **1** adopted a different orientation. The linker amine positioned itself directly above the heme propionates and established electrostatic interactions with these groups. Meanwhile, the tail group occupied a serine-capped peripheral pocket (S607) and remained distant (~ 9 Å) from D602, which correlated well with the enzymatic assays where **2** achieved 1075-fold selectivity, while **1** only achieved 337-fold selectivity over heNOS. This indicates that tail-group positioning directly influences isoform selectivity. Another factor contributing to the poor selectivity of **1** is its hydrophobic tail, which fits well into the heNOS peripheral pocket formed by the bulky Phe105 residue. In contrast, the corresponding His342 in hnNOS, located only 3–4 Å from the tail group of **1**, favors a slightly polar tail, limiting favorable interactions.

Motivated by these observations, we designed compounds **3** and **4** incorporating a dimethylamino-substituted tail group to establish more favorable peripheral pocket interactions in hnNOS. The dimethylamino moiety provides cationic character capable of engaging aromatic residues within the hnNOS peripheral pocket through electrostatic and cation-π interactions, while the ethylene linker offers flexibility for optimal binding geometry. This design strategy maintains the potency-determining head-group anchoring while exploiting the isoform-specific steric differences to enhance selectivity. Furthermore, we conducted NOS enzymatic assays to evaluate NOS inhibition and performed PAMPA assays to assess potential blood-brain barrier permeability. Molecular dynamics simulations and free energy calculations were subsequently performed to elucidate the binding modes and mechanistic basis for the observed selectivity.

### Chemistry

**Scheme 1:**
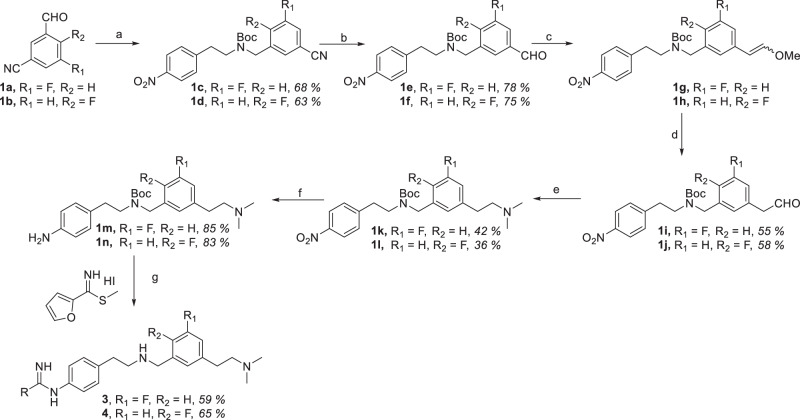
Synthesis of **3** and **4**. (a) (i) 2-(4-Nitrophenyl)ethan-1-amine, NaBH_3_CN, cat. glacial AcOH, THF, 0 °C to rt, 2 h (ii) (Boc)_2_O, NaHCO_3_, rt, 1 h; (b) DIBAL, PhMe, −78 °C, 1 h; (c) CH_3_OCH_2_PPh_3_Cl, *t*-BuOK, 0 °C to rt, 2 h; (d) Hg(OAc)_2_, THF:H_2_O(10:1) 0 °C, 1 h; (e) dimethylamine, NaBH_3_CN, cat. glacial AcOH, THF, 0 °C to rt, 2 h; (f) Pd/C, H_2_, MeOH, rt, 24 h; (g) (i) EtOH, rt, 24 h; (ii) HCl (3 M in MeOH), MeOH, rt, 12 h.

To access compounds **3** and **4**, the synthetic route shown in Scheme 1 was employed. Reductive amination of 2-(4-nitrophenyl)ethan-1-amine with 4-fluoro-3-formylbenzonitrile (**1a**) and 3-fluoro-5-formylbenzonitrile (**1b**) in the presence of catalytic AcOH and NaBH_3_CN provided the corresponding secondary amines, which were subsequently protected with Boc anhydride to afford intermediates **1c** and **1d**, respectively [18]. Selective reduction of the nitrile moieties with DIBAL at −78 °C furnished the corresponding aldehydes **1e** and **1f**, setting the stage for controlled one-carbon chain extension [19]. Wittig olefination using methoxymethyltriphenylphosphonium chloride and *t*-BuOK produced the vinyl ether intermediates **1g** and **1h**, which were smoothly hydrolyzed with Hg(OAc)₂ to aldehydes **1i** and **1j** [20]. This two-step homologation sequence provided a modular handle for subsequent tail-group modification. Reductive amination of aldehydes **1i** and **1j** with dimethylamine under mild conditions yielded tertiary amines **1k** and **1l**, introducing the terminal cationic functionality designed to engage in electrostatic and cation–π interactions within the NOS peripheral pocket. The nitro groups of these intermediates were then efficiently reduced by catalytic hydrogenation (Pd/C, H₂) to furnish anilines **1m** and **1n**, enabling subsequent installation of the heterocyclic head group [16]. Final coupling of intermediates **1m** and **1n** with methyl furan-2-carbimidothioate hydrogen iodide salt in ethanol afforded the corresponding intermediates [16]. Acidic deprotection (3 M HCl in MeOH) removed the Boc protecting group and furnished the target inhibitors **3** and **4** as their hydrochloride salts.

### Enzyme activity of newly synthesized nNOS inhibitors

All biochemical assays were conducted using the hydrochloride salts of synthesized compounds. Inhibitory potency and isoform selectivity were evaluated using a NO hemoglobin capture assay [21] (see Supporting Information for experimental details) with results summarized in Table 1. Compounds were screened against both rat and human nNOS to assess preclinical and clinical relevance, with a human-to-rat potency ratio (*K*_i_ hn/rn) near unity being preferred. Membrane permeability was assessed using the parallel artificial membrane permeability assay for the blood-brain barrier (PAMPA-BBB) on lead compounds with results summarized in Table 2.

**Table 1:** *K*_i_ values and selectivities of novel synthesized molecules

Compound	*K*_i_ (nM)^*a*^				Selectivity ^*b*^		
	rat nNOS (rn)	human nNOS (hn)	human iNOS (hi)	human eNOS (he)	hn/rn	hn/hi	hn/he
1•3HCl	5	20	1215	6735	4	61	337
2•3HCl	15	19	2181	20423	1.3	115	1075
3•3HCl	22	16	3664	27709	0.7	229	1732
4•3HCl	22	18	5204	27465	0.8	289	1526

^*a*^*K*_i_ values were calculated from the IC_50_ values of the corresponding dose-response curves using the Cheng-Prusoff equation. For each compound, 8–11 concentrations were tested, and the IC_50_ value was calculated from an average of at least two duplicates. All standard errors were less than 5%

^*b*^Selectivity values were determined by calculating the ratios of respective *K*_i_ values. The ratio hn/rn is desired to be as close to 1.0 as possible to avoid significant differences between rat and human doses for clinical studies. For hn/hi and hn/he ratios, higher values are favorable. hn is human nNOS, rn is rat nNOS, hi is human iNOS, he is human eNOS

The previously reported inhibitors **1** and **2** exhibited strong potency toward hnNOS, with *K*_i_ values of 20 and 19 nM, respectively. The newly designed inhibitors **3** and **4** also displayed potent hnNOS inhibition (*K*_i_ = 16 and 18 nM, respectively), indicating that incorporation of the modified tail amine did not compromise binding affinity. Notably, both compounds showed comparable potency toward rat and human nNOS, with hn/rn ratios close to unity (0.7 for **3** and 0.8 for **4**, i.e., more potent with hnNOS than rnNOS), supporting their translational relevance. More importantly, both compounds showed substantially enhanced isoform selectivity relative to the parent scaffolds. Compound **3** exhibited 229-fold selectivity over hiNOS and 1731-fold selectivity over heNOS, while compound **4** demonstrated 289-fold selectivity over hiNOS and 1526-fold selectivity over heNOS. Collectively, these results indicate that the flexible cationic tail preserved high hnNOS potency while markedly improving selectivity over the endothelial and inducible isoforms.

### Binding modes of 4 in hnNOS and heNOS

**Fig. 3:**
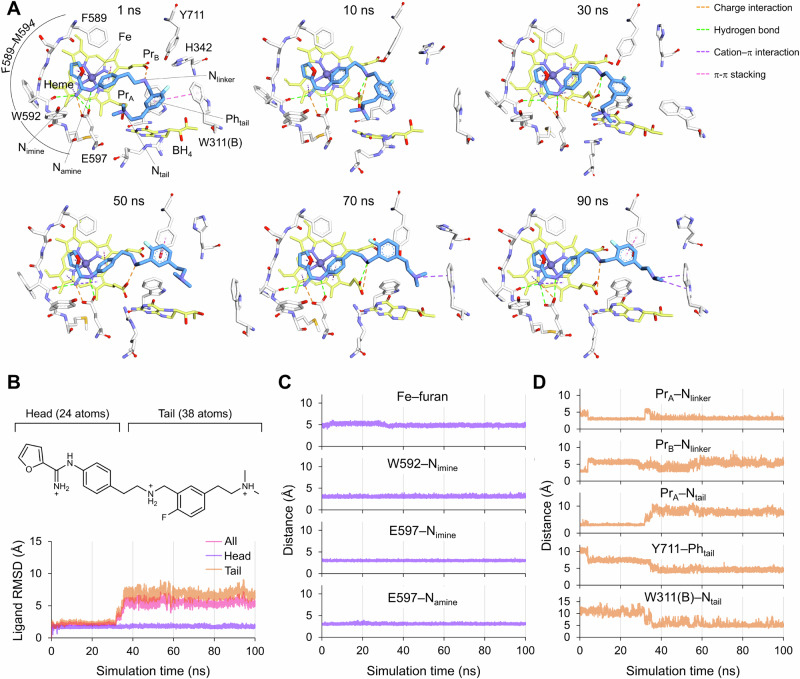
MD simulation results of the hnNOS-**4** complex. (**A**) Binding modes of **4** in representative frames at the indicated time points. White, yellow, and blue sticks represent hnNOS active site residues, cofactor atoms, and **4**, respectively. (**B**) RMSD of all atoms (All), head group (Head), and tail group (Tail) of **4**. (**C,** **D**) Distances from hnNOS active site atoms to head (**C**) and tail group atoms (**D**). Fe, heme iron; N_imine_, nitrogen atom of carboximidamide iminium; N_amine_, nitrogen atom of carboximidamide amide; Pr_A_, heme propionate A; Pr_B_, heme propionate B; N_linker_, nitrogen atom of linker amine; N_tail_, nitrogen atom of tail group terminal amine; Ph_tail_, tail group phenyl ring centroid.

To elucidate the binding modes of **4** in hnNOS, a 100 ns molecular dynamics (MD) simulation was conducted (Fig. 3). The initial structure of the hnNOS-**4** complex was generated by docking-based prediction using the crystal structure of the hnNOS-**1** complex (PDB 9MWA) [16]. Throughout the simulation (Fig. 3A), the head group of **4** maintained a stable conformation based on the head group RMSD (Fig. 3B) and consistent interactions (Fe–furan distance = ~5.0 Å and W592–N_imine_, E597–N_imine_, E597–N_amine_ distances = ~3.0 Å; Fig. 3C), which include π-interactions with the heme iron and pyrrole rings, as well as hydrogen bonds and charge interactions with the W592 main chain carbonyl and the E597 side chain carboxylate (Fig. 3A).

In contrast, the tail group exhibited substantial motion after ~31 ns of simulation, as indicated by markedly high tail group RMSD (Tail in Fig. 3B). This conformational transition resulted in the disruption of the charge interaction between the terminal amine and heme propionate A, which had existed for the first ~31 ns of simulation, as monitored by increased Pr_A_–N_tail_ distance (from ~3.0 Å to > 6.0 Å; Fig. 3D). However, the linker amino group maintained its charge interaction with the heme propionate (Pr_A_–N_linker_ distance = ~3.0 Å; Fig. 3D). Following this transition, the tail group migrated to the peripheral pocket of the active site, forming π-π stacking with the side chain of Y711 and/or a cation-π interaction with the W311(B) side chain indole (Y711–Ph_tail_ distance = ~4.5 Å and W311(B)–N_tail_ distance = 4.0–6.5 Å; Fig. 3D). This interaction pattern is distinct from that of the inhibitor **2** tail group, which binds to the heme propionate A and D602 side chain (for the binding mode, see PDB 8FGG).

**Fig. 4:**
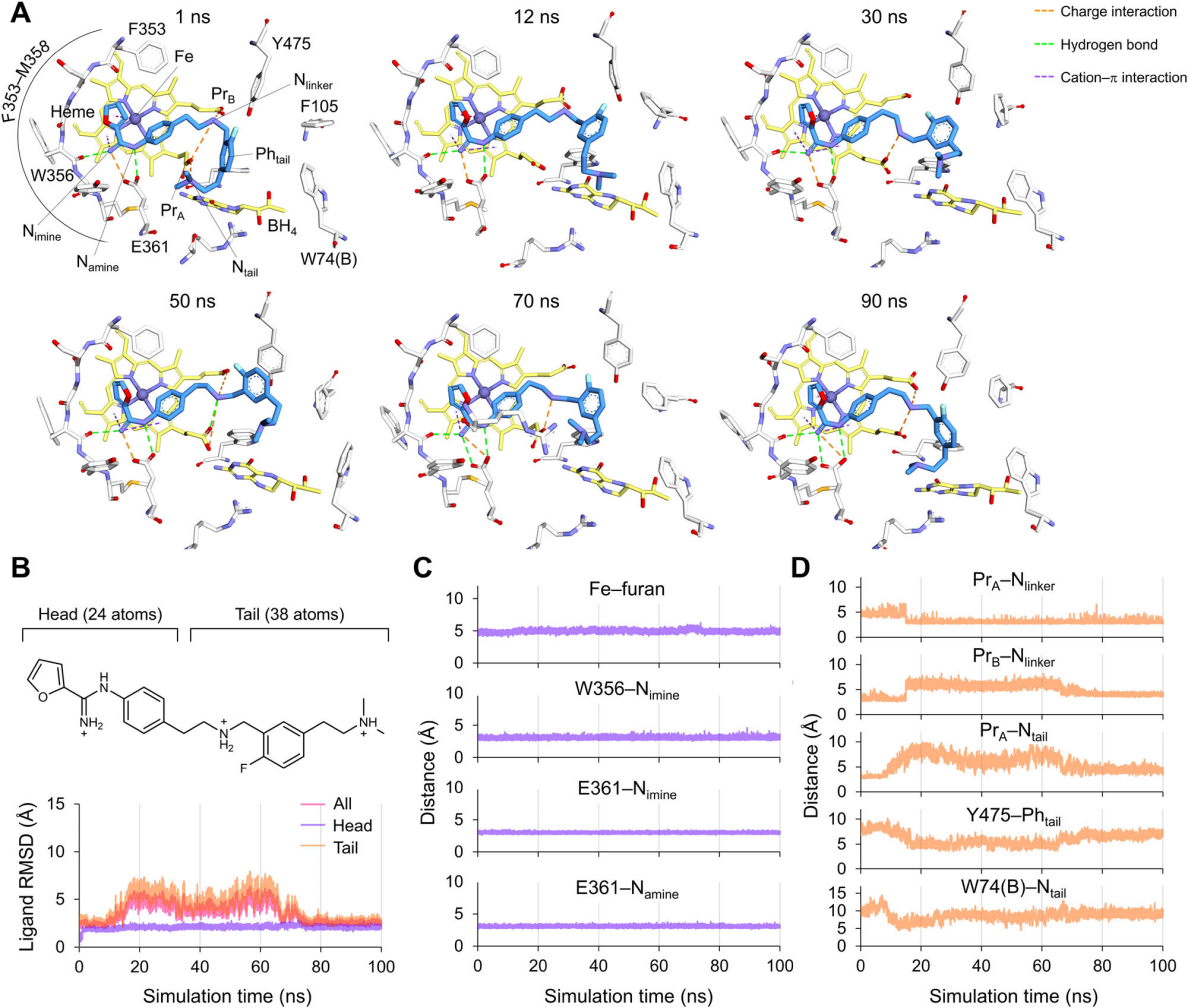
MD simulation results of the heNOS-**4** complex. (**A**) Binding modes of **4** in representative frames at the indicated time points. White, yellow, and blue sticks represent heNOS active site residues, cofactor atoms, and **4**, respectively. (**B**) RMSD of all atoms (All), head group (Head), and tail group (Tail) of **4**. (**C,** **D**) Distances from heNOS active site atoms to head (**C**) and tail group atoms (**D**). Fe, heme iron; N_imine_, nitrogen atom of carboximidamide iminium; N_amine_, nitrogen atom of carboximidamide amide; Pr_A_, heme propionate A; Pr_B_, heme propionate B; N_linker_, nitrogen atom of linker amine; N_tail_, nitrogen atom of tail group terminal amine; Ph_tail_, tail group phenyl ring centroid.

To further investigate the hn/heNOS selectivity of **4**, the binding mode of **4** in the heNOS active site was also simulated with the same approach using the crystal structure of the heNOS-**1** complex (PDB ID: 9MWN) [17] (Fig. 4). Similar to hnNOS, the head group of **4** retained a stable conformation and interaction with the heNOS active site atoms (Fig. 4A–4C). However, except for the interaction between the linker amino group and heme propionates, the tail group drifted around the peripheral pocket without forming any stable interactions during the simulation (Fig. 4B, D). This may result from the heNOS-specific residue F105, the structural equivalent of hnNOS H342. Since this relatively bulky side chain is located between the **4** head group and W74(B), the formation of a cation-π interaction between the terminal amine and W74(B) could be sterically hindered. Mutagenesis of F105 into a less bulky residue, such as histidine, could further support this computational interpretation by experimentally assessing whether reducing steric bulk mitigates the hn/heNOS selectivity of **4**.

The difference in binding affinity, possibly from the distinct interactions of the tail group of **4** with hnNOS and heNOS, was quantitatively analyzed by running MM-GBSA calculations on the MD trajectories. The MD simulation trajectory of the hnNOS-**4** complex was divided into two different parts to compare the theoretical binding free energy (*ΔG*_Bind_) of two distinct binding modes with (40–100 ns) and without (0–30 ns) the W311(B) cation-π interaction. Frames with the cation-π interaction yielded a more favorable *ΔG*_Bind_ of −67.5 ± 0.5 kcal/mol compared to −58.6 ± 0.8 kcal/mol without the interaction, indicating an energetic gain of ~8.9 kcal/mol. This value was also comparable to the *ΔG*_Bind_ calculated from the previously reported [9] 100 ns MD trajectories for the hnNOS-**4** complex (−69.5 ± 0.4 kcal/mol), consistent with the similar hnNOS binding affinities of the two inhibitors (*K*_i_ = 20 and 18 nM for **1** and **4**, respectively). In addition, the *ΔG*_Bind_ calculated from the heNOS-**4** trajectory (−62.0 ± 0.4 kcal/mol) was ~5.5 kcal/mol less favorable, reinforcing that the particularly strong interaction between cationic ammonium and indole ring, as widely recognized in biological model systems, may mainly contribute to the hn/heNOS selectivity of **4** [22].

### Effect of fluorine substitution on potency and selectivity

Compounds **3** and **4** differ only in the position of the fluorine substituent on the benzyl tail phenyl ring (meta vs ortho). This regio isomeric variation had no measurable impact on hnNOS potency or isoform selectivity, indicating that fluorine placement at this position is well tolerated by the binding pocket and does not directly contribute to target engagement. Notably, incorporation of a fluorine substituent on the aryl tail is expected to attenuate oxidative metabolism at the phenyl ring [23, 24], suggesting that this modification may primarily serve to enhance metabolic stability and improve pharmacokinetic properties without compromising the nNOS activity.

### Assessment of permeability with the PAMPA-BBB Assay

Compounds **3** and **4** were evaluated for blood-brain barrier (BBB) permeability using the PAMPA-BBB assay alongside parent compounds **1** and **2**. The newly designed inhibitors **3** and **4** exhibited moderate BBB permeability with *P*_e_ values of 3.98 × 10⁻⁶ and 4.21 × 10⁻⁶ cm s⁻¹, respectively, which was attributed to the introduction of the polar secondary amine linker and cationic tertiary amine tail group. In contrast, the previously reported compounds **1** and **2** demonstrated superior BBB penetration, with *P*_e_ values of 7.52 × 10⁻⁶ and 13.7 × 10⁻⁶ cm s⁻¹, respectively, likely due to their more lipophilic structural features. Although compounds **3** and **4** showed significantly improved isoform selectivity compared to **1** and **2**, this selectivity gain was offset by a modest reduction in BBB permeability. Nevertheless, both **3** and **4** retained sufficient BBB penetration (CNS + ) to support neurological applications, suggesting that further optimization of the tail group polarity could enhance membrane permeability while maintaining the selectivity advantages of the current design (Table 2).

**Table 2:** Effective permeability (*P*_e_) in the PAMPA − BBB assay

Compound	Reported *P*_e_ (10^−6 ^cm s^−1^)	Determined *P*_e_^*a*^ (10^−6 ^cm s^-1^)	Prediction^*b*^
( ± )-Verapamil	16	20.1 ± 1.26	CNS (+)
Theophylline	0.12	0.08 ± 0.04	CNS (-)
**1**		7.52 ± 0.44	CNS (+)
**2**		13.7 ± 0.64	CNS (+)
**3**		3.98 ± 0.32	CNS (+)
**4**		4.21 ± 0.38	CNS (+)

^a^Effective permeability values were obtained in our in-house conditions. All assays were in triplicate for each compound at 200 μM concentration over 17 h (See SI)

^b^CNS (+) = likely high BBB permeation. CNS (−) = likely low BBB permeation

## Conclusions

In this study, we employed a hybrid structure-based design strategy to develop compounds **3** and **4**, which maintained the potent hnNOS inhibitory activity of the parent scaffolds while achieving substantially enhanced isoform selectivity. NOS enzymatic assays demonstrated that **3** and **4** retained sub-20 nM potency against hnNOS with improved selectivity over heNOS ( > 1500-fold) and hiNOS ( > 200-fold), representing significant improvements over lead compounds **1** and **2**. Molecular dynamics simulations suggested that this selectivity may arise from a dynamically formed cation-π interaction between the dimethylamino tail group and W311(B) in the hnNOS peripheral pocket, an interaction that is sterically precluded in heNOS by the bulky F105 residue. MM-GBSA free energy calculations quantified this interaction as contributing approximately 8.9 kcal/mol to binding affinity, with a corresponding 5.5 kcal/mol selectivity penalty in heNOS. These findings suggest that peripheral pocket interactions, rather than conserved catalytic site residues, are the primary drivers of isoform selectivity in this inhibitor class.

While compounds **3** and **4** exhibited reduced BBB permeability compared to **1** and **2**, both retained sufficient membrane penetration (CNS + ) to support neurological applications. The selectivity-permeability trade-off observed here highlights an important design consideration for future inhibitor optimization. Further structural modifications targeting the tail group polarity and lipophilicity may enable the development of compounds with both superior selectivity and enhanced CNS bioavailability, thereby advancing the therapeutic potential of hnNOS inhibitors for neurological disorders and melanoma.

**Supporting Information:** Detailed protocols for NOS enzyme inhibition assays and PAMPA-BBB permeability measurements; computational details for molecular dynamics simulations and MM-GBSA free energy calculations; complete synthetic procedures and characterization data (^1^H NMR, ^13^C NMR, LCMS) for final compounds **3** and **4**.

## Supplementary information

Supplementary Information

## Data Availability

The data supporting the findings of this study are available within the article and its Supporting Information. Detailed protocols for NOS inhibition assays and PAMPA-BBB measurements, computational details for molecular dynamics simulations and MM-GBSA free energy calculations, and complete synthetic procedures and characterization data (¹H NMR, ¹³C NMR, LCMS) are provided. Additional data are available from the corresponding author upon reasonable request.
