# Supplementary material for: Dynamic tail amine interactions drive isoform selectivity for potent human neuronal nitric oxide synthase inhibitors
Source: Med Chem Res. 2026 Apr 10;35(4):757–64. doi: 10.1007/s00044-026-03552-3 (PMC13128701; doi:10.1007/s00044-026-03552-3)
Supplement: Supplementary file 1 — Supplementary Information [file 44_2026_3552_MOESM1_ESM.docx]

**Supporting Information**

**Dynamic Tail Amine Interactions Drive Isoform Selectivity for Potent Human Neuronal Nitric Oxide Synthase Inhibitors**

Amardeep Awasthi^1^, Koon Mook Kang^1^, and Richard B. Silverman^*, 1, 2, 3^

^1^ Department of Chemistry, Chemistry of Life Processes Institute, Center for Developmental Therapeutics, Northwestern University, 2145 Sheridan Road, Evanston, Illinois 60208-3113, United States

^2^ Department of Molecular Biosciences, Northwestern University, Evanston, Illinois 60208

^3^ Department of Pharmacology, Feinberg School of Medicine, Northwestern University, Chicago, Illinois 60611, United States

*Corresponding author:

**Richard B. Silverman** - Department of Chemistry, Department of Molecular Biosciences, Chemistry of Life Processes Institute, Center for Developmental Therapeutics, Northwestern University, 2145 Sheridan Road, Evanston, Illinois 60208-3113, United States; <http://orcid.org/0000-0001-9034-1084> ;Email: [Agman@chem.northwestern.edu](mailto:Agman@chem.northwestern.edu) Fax: +1 847 491 7713

**Authors:**

**Amardeep Awasthi -**Department of Chemistry, Chemistry of Life Processes Institute, Center for Developmental Therapeutics, Northwestern University, Evanston, Illinois 60208-3113, United States

**Koon Mook Kang-**Department of Chemistry, Chemistry of Life Processes Institute, Center for Developmental Therapeutics, Northwestern University, Evanston, Illinois 60208-3113, United States

**Table of Contents:**

| S. No |  | Pages |
| --- | --- | --- |
| 1 | NOS Enzyme Inhibition Assay Protocol | S3 |
| 2 | PAMPA BBB assay Protocol | S3-5 |
| 3 | Dose response curves | S5-6 |
| 4 | Computational Details | S6-7 |
| 5 | Experimental procedures | S7-10 |
| 6 | Copies of ^1^H NMR and ^13^C NMR | S11-12 |
| 7 | References | S13-15 |

1. **NOS Enzyme Inhibition Assay Protocol**

The NOS inhibitory activity of **1-21** was measured by the hemoglobin (Hb) NO capture assay following a protocol described previously.^1-2^ The production of NO was monitored by the rapid oxidation of oxyhemoglobin (oxyHb) to methemoglobin (metHb) by NO.^1^


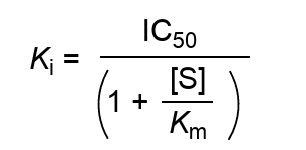
Purified recombinant full-length NOSs, including rat nNOS (rnNOS),^3-4^ human nNOS (hnNOS),^5^ human iNOS (hiNOS),^6^ and human eNOS (heNOS),^5^ were used in activity assays. These proteins were expressed in *Escherichia coli* and purified as described previously.^3-6^ Activity assays were performed in 100 mM HEPES (4-(2-hydroxyethyl)-1-piperazine-ethanesulfonic acid) buffer with 10% glycerol (pH 7.4-7.5) at 37 °C in the presence of 10 *μ*M L-Arg, 10 *μ*M H_4_B, 100 *μ*M NADPH, 0.83 mM CaCl_2_, 320 units/mL calmodulin, and 3 *μ*M human oxyhemoglobin. A concentration of L-Arg of 10 *μ*M was used as it does not cause NOS uncoupling and is close to the *K*_m_ values of all three NOS isoforms so that competitive inhibitors can be detected effectively. In the case of iNOS, CaCl_2_ and calmodulin were omitted and replaced by HEPES buffer (100 mM, 10 % glycerol, pH 7.4-7.5) because iNOS activation is calcium-independent. The assay was performed in 96-well plates using a Biotek Gen5 microplate reader, with NOS enzymes and hemoglobin dispensed automatically by the plate reader. NO production was kinetically monitored at 401 nm for 6 min. The inhibition constants (*K*_i_) for all NOSs were calculated from the IC_50_ values of the dose-response curves using the Cheng-Prusoff equation,^7^

where *K*_m_ is the Michaelis constant: *K*_m_ _(hnNOS)_ = 1.6 *μ*M; *K*_m_ _(rnNOS)_ = 1.3 *μ*M; *K*_m_ _(hiNOS)_ = 8 *μ*M; *K*_m_ _(heNOS)_ = 3.9 *μ*M.^8^ Dose−response curves were constructed from ten to eleven test concentrations (10 mM to 10 nM), and IC_50_ values were calculated by nonlinear regression using GraphPad Prism software. The calculated standard deviations from dose−response curves of the assays were less than 10% with all NOSs.

**2. PAMPA-BBB Assay Protocol**

Blood-brain barrier penetration was estimated using the PAMPA−BBB assay, in which a porcine brain lipid (PBL) was used as an artificial membrane.^9-11^ The five commercial drugs, (±)-verapamil, desipramine, chlorpromazine, dopamine, and theophylline; phosphate buffer saline (PBS, 10 mM); DMSO (for biology); and dodecane (analytical standard) were purchased from Sigma-Aldrich. The porcine brain lipid (PBL) was obtained from Avanti Polar Lipids (100 mg, powder, catalogue no. 141101P). The donor plate used in the assay is a 96-well filter plate with a hydrophobic polyvinylidene fluoride (PVDF) membrane (pore size 0.45 μm, nonsterile, catalogue no. MAIPNTR10), and the acceptor plate is a 96-well transport receiver plate (catalogue no. MATRNPS50), both from Millipore Sigma. A 96-well UV plate with a flat bottom obtained from Greiner Bio- One was used for UV measurements (catalogue no. 655801). Test compounds were first dissolved in DMSO to make a 10 mM stock solution. Then 40 μL of the stock solution was diluted with 1960 μL of 10 mM PBS buffer (pH 7.5) to generate a final concentration of 200 μM (2% DMSO). The acceptor plate was filled with 250 μL of 10 mM PBS (2% DMSO, pH 7.5). The donor plate was first coated with 4 μL of PBL (20 mg/mL in dodecane), then 250 μL of a test compound (200 μM) was added to the donor plate. Each test compound was measured in triplicate. The donor plate was then carefully placed on top of the acceptor plate to make a “sandwich”, which was incubated at 25 °C for 17 h in a saturated humidity atmosphere with an orbital agitation at 100 rpm. During this time, compounds diffuse from the donor plate to the acceptor plate. After incubation, 150 μL of test solution was taken from each well from both donor and acceptor plates and transferred to the UV plate for measurement. The concentration of a compound in each donor and acceptor well was determined by using a standard curve, which was built from its UV absorbance at λmax of various concentrations (1−200 μM). The effective permeability (*P*_e_) was calculated using the following equation^12^,


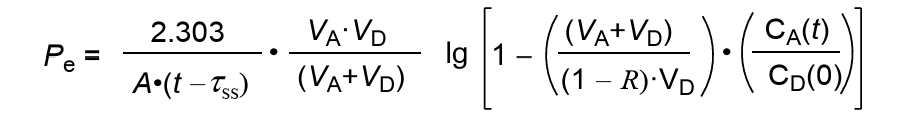


where *P*_e_ is the effective permeability (cm s−1); VA and VD are the volume of the acceptor and donor wells (0.25 cm^3^), respectively; CA (t) is the concentration of the acceptor well at time t; CD(0) and CD(t) are the concentrations of the donor well at t0 and t, respectively; A is the filter well area (0.21 cm2); t is the incubation time (s); τss is the time to reach a S4 steady state (usually very short compared with the incubation time); and R is the retention membrane factor, which was calculated using the following equation:


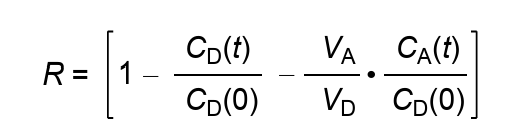


*P*_e_ is reported as an average of triplicates with a standard deviation.

1. **Dose Response Curves**

|  |  |
| --- | --- |
|  |  |
|  |  |
|  |  |

1. **Computational Details**

To model the complexes of **4** with hnNOS and heNOS, molecular docking was conducted using Glide XP^13,14^ in the Schrödinger Release 2024-4 suite. The crystal structures of hnNOS (PDB 9MWA) and heNOS (PDB 9MWN) were retrieved from the RCSB Protein Data Bank (https://www.rcsb.org).^15^ The structures were prepared using the Protein Preparation Wizard^16^. Missing loops were modeled using Prime^17^ as follows: residues 345–351 of 9MWA chain B were filled with QHARRPE; residues 107–121 of 9MWN chain A with RKLQGRPSPGPPAP; and residues 107–118 of 9MWN chain B with RKLQGRPSPGPP. Each structure was subsequently minimized in Prime to relieve steric clashes and unfavorable interactions using the ‘Automatic’ option, which combines conjugate gradient and truncated Newton methods, with an RMS gradient convergence threshold of 0.01 kcal/mol/Å. The preprocessed structures were then used to generate grids in Glide after removing residual solvent and ion atoms. All structures were parametrized with the OPLS4 force field.^18^ The ligand structure was drawn in ChemDraw 25.5 (Revvity; Waltham, MA, USA) and imported into Maestro 2024-4 (Schrödinger; New York, NY, USA). The ligand preparation included manual assignment of cationic charges to the carboximidamide imine, linker amine, and terminal amine groups, followed by LigPrep processing. During docking, the coordinates of the head group atoms from the minimized crystal structures were applied as the core constraint to retain the head group binding mode. The resulting **4**-binding complexes were then minimized as described above, then solvated in an orthorhombic box of SPC water with a minimum 10 Å buffer between the complex and the box boundary. Na^+^ and Cl^–^ ions were added to achieve a physiological ionic strength of 0.15 M NaCl and neutralize the system using the System Builder in Desmond (D. E. Shaw Research, New York, NY, USA).^19^ Each system was equilibrated using the standard 5-step relaxation protocol provided by Desmond, followed by a production MD simulation under the NPT ensemble (Nose-Hoover chain thermostat and Martyna-Tobias-Klein barostat)^20,21^ at 300 K for 100 ns. MD trajectories and energy profiles were recorded every 5 ps and analyzed using the Maestro interface and Desmond module. The theoretical binding free energy was estimated from each trajectory using the *thermal_mmgbsa* module, with frames sampled every 100 snapshots (~0.5 ns). The trajectory for the hnNOS-**1** complex was obtained from MD simulations reported previously^22^.

1. **Experimental Procedures**

**Chemistry**

Chemical reagents were obtained from commercial sources (Sigma-Aldrich, TCI, Combi-Blocks, and AA Blocks) and used without additional purification. Anhydrous solvents (THF, acetonitrile, and DMF) were purchased from Thermo Fisher and dried using a solvent purification system prior to use. All reactions were conducted under a nitrogen atmosphere. Thin-layer chromatography (TLC) was performed on Merck silica gel 60 F254 plates (0.25 mm thickness) with visualization under UV light (254 nm). Flash column chromatography was carried out on a Teledyne CombiFlash Nextgen 300+ instrument equipped with prepacked silica columns.

^1^H and ^13^C NMR spectra were recorded on a Bruker Avance III spectrometer operating at 500 and 125 MHz, respectively, using CDCl_3_, CD_3_OD, DMSO-d_6_, or D₂O as solvents. Chemical shifts (δ) are reported in parts per million relative to internal solvent standards, with coupling constants (J) expressed in hertz. Multiplicities are designated as s (singlet), d (doublet), t (triplet), q (quartet), sep (septet), dd (doublet of doublets), dt (doublet of triplets), m (multiplet), and br (broad). High-resolution mass spectra were obtained on an LC-TOF instrument (Agilent G1312) operating in positive-ion electrospray ionization (ESI) mode. The purity of all final compounds (3 and 4) was assessed by LC-MS and was ≥ 95%.

**General Procedure 1: Reductive Amination and Boc Protection**

A solution of the amine intermediate (1.0 equiv) and the corresponding benzaldehyde (1.1 equiv) in anhydrous THF (5 mL) was treated with glacial acetic acid (0.1 equiv) and stirred at room temperature for 1 h. Sodium cyanoborohydride (1.1 equiv) was added portionwise over 10 min, and the reaction was allowed to proceed overnight at ambient temperature. Methanol (2 mL) was added dropwise to quench the reaction, and the mixture was concentrated under reduced pressure. The residue was dissolved in ethyl acetate (15 mL) and sequentially washed with aqueous sodium hydroxide (1 M, 5 mL) and brine (20 mL). The organic layer was dried over anhydrous sodium sulfate, filtered, and concentrated to afford the crude secondary amine as a viscous orange oil. To the crude product were added di-tert-butyl dicarbonate (1.5 equiv), sodium bicarbonate (1.2 equiv), and anhydrous acetonitrile (5 mL). After stirring at room temperature for 15 min, the reaction mixture was concentrated in *vacuo*. The residue was taken up in ethyl acetate (50 mL), washed with brine (30 mL), dried over anhydrous sodium sulfate, and concentrated. The crude material was purified by flash column chromatography to afford the Boc-protected intermediates.

**General Procedure 2: Wittig Olefination and Vinyl Ether Hydrolysis**

A round-bottom flask was charged with methoxymethyltriphenylphosphonium chloride (2.5 equiv) and potassium tert-butoxide (2.3 equiv) under a nitrogen atmosphere. Anhydrous THF (10 mL) was added, and the mixture was stirred at 0 °C for 30 min to generate the ylide. The aldehyde intermediate (1.0 equiv) was dissolved in the minimum amount of anhydrous THF and added slowly to the ylide solution. Reaction progress was monitored by TLC, and upon completion, the mixture was quenched with saturated aqueous ammonium chloride (10 mL). The reaction mixture was extracted with ethyl acetate (2 × 15 mL), and the combined organic layers were concentrated under reduced pressure to afford the crude vinyl ether intermediate. The crude vinyl ether (1.0 equiv) was dissolved in a THF:water mixture (10:1 v/v, 11 mL) and cooled to 0 °C. Mercury(II) acetate (3.0 equiv) was added, and the reaction was stirred at 0 °C for 1 h. The reaction was quenched with saturated aqueous sodium thiosulfate (10 mL), and the suspension was filtered through Celite. The filtrate was diluted with ethyl acetate (15 mL) and washed sequentially with aqueous sodium chloride (20 mL) and water (10 mL). The organic layer was dried over anhydrous sodium sulfate, filtered, and concentrated under reduced pressure. Purification by flash column chromatography afforded the corresponding aldehyde intermediates.

**General Procedure 3: Head Group Installation and Hydrochloride Salt Formation**

The amine intermediate (1.0 equiv) was dissolved in absolute ethanol (2 mL), and the methyl furan-2-carbimidothioate hydroiodide salt (2.0 equiv) was added. The reaction mixture was stirred at room temperature for 24 h and then concentrated under reduced pressure. Purification of the crude residue was performed and was further dissolved in 3 M hydrochloric acid in methanol (1 mL) and stirred at room temperature for 24 h, then concentrated in *vacuo*. Anhydrous methanol (0.2 mL) was added to the residue, and the mixture was gently warmed (40 °C water bath) until complete dissolution was achieved. Anhydrous diethyl ether (1 mL) was added dropwise until precipitation occurred. The resulting solid was isolated by decantation, washed with ice-cold anhydrous diethyl ether (3 × 5 mL portions), and dried under reduced pressure to furnish compounds **3** and **4** as their trihydrochloride salts.

***N*-(4-(2-((5-(2-(Dimethylamino)ethyl)-3-fluorobenzyl)amino)ethyl)phenyl)furan-2-carboximidamide hydrochloride salt (3)**

^1^H NMR (500 MHz, D_2_O) *δ* 7.79 (d, *J* = 1.7 Hz, 1H), 7.45 (d, *J* = 3.8 Hz, 1H), 7.37 – 7.23 (m, 4H), 7.10 (s, 1H), 7.05 (ddt, *J* = 13.6, 9.3, 2.2 Hz, 2H), 6.67 (dd, *J* = 3.8, 1.8 Hz, 1H), 4.14 (s, 2H), 3.31 (dd, *J* = 9.4, 6.6 Hz, 2H), 3.00 – 2.96 (m, 4H), 2.79 (s, 6H). ^13^C NMR (125 MHz, D_2_O) *δ* 163.7, 161.7, 152.4, 148.9, 140.6, 139.6 (d, *J* = 8.2 Hz), 137.5, 133.2 (d, *J* = 8.2 Hz), 132.0, 126.2 (d, *J* = 3.9 Hz), 119.0, 116.9 (d, *J* = 21.7 Hz), 115.5 (d, *J* = 22.5 Hz), 113.3, 57.8, 50.2, 47.7, 42.8, 31.2, 29.8. **LRMS** (ESI) calcd for C_24_H_30_FN_4_O [(M + H)^+^], 409.24; found, 409.12.

***N*-(4-(2-((5-(2-(Dimethylamino)ethyl)-2-fluorobenzyl)amino)ethyl)phenyl)furan-2-carboximidamide hydrochloride salt (4)**

^1^H NMR (500 MHz, D_2_O) *δ* 7.88 – 7.84 (m, 1H), 7.52 (s, 1H), 7.42 (d, *J* = 8.0 Hz, 3H), 7.36 (d, *J* = 8.1 Hz, 2H), 7.16 (d, *J* = 9.2 Hz, 2H), 6.75 (dd, *J* = 4.2, 1.9 Hz, 1H), 4.28 (s, 2H), 3.42 – 3.36 (m, 2H), 3.35 – 3.30 (m, 2H), 3.06 (q, *J* = 7.4 Hz, 4H), 2.86 (d, *J* = 0.8 Hz, 6H). ^13^C NMR (125 MHz, D_2_O) *δ* 162.2, 160.2, 152.4, 148.8, 140.8 (d, *J* = 8.4 Hz), 140.6, 137.5, 132.6 (d, *J* = 4.1 Hz), 130.6, 126.2, 125.3 (d, *J* = 3.8 Hz), 118.9, 116.6 (d, *J* = 15.7 Hz), 116.2 (d, *J* = 22.0 Hz), 113.3, 57.7, 47.7, 44.3, 42.8, 31.1, 29.7. **LRMS** (ESI) calcd for C_24_H_30_FN_4_O [(M + H)^+^], 409.24; found, 409.16.

1. **^1^H NMR and ^13^C NMR of compound 3**

**^1^H NMR and ^13^C NMR of compound 4**

1. **References**
2. Hevel JM, Marletta MA. Nitric‑oxide synthase assays. Methods Enzymol. 1994;233:250–258. [**https://doi.org/10.1016/S0076-6879(94)33028-X.[1**](https://doi.org/10.1016/S0076-6879(94)33028-X.%5B1)]
3. Do HT, Wang H‑Y, Li H, Chreifi G, Poulos TL, Silverman RB. Improvement of cell permeability of human neuronal nitric oxide synthase inhibitors using potent and selective 2‑aminopyridine‑based scaffolds with a fluorobenzene linker. J Med Chem. 2017;60:9360–9375. [**https://doi.org/10.1021/acs.jmedchem.7b01245.**](https://doi.org/10.1021/acs.jmedchem.7b01245.)​
4. Roman LJ, Sheta EA, Martasek P, Gross SS, Liu Q, Masters BSS. High level expression of functional rat neuronal nitric oxide synthase in Escherichia coli. Proc Natl Acad Sci USA. 1995;92:8428–8432. [**https://doi.org/10.1073/pnas.92.18.8428.**](https://doi.org/10.1073/pnas.92.18.8428.)​
5. Li H, Shimizu H, Flinspach M, Jamal J, Yang W, Xian M, Cai T, Wen EZ, Jia Q, Wang PG, Poulos TL. The novel binding mode of N‑alkyl‑N'‑hydroxy guanidine to neuronal nitric oxide synthase provides mechanistic insights into NO biosynthesis. Biochemistry. 2002;41:13868–13875. [**https://doi.org/10.1021/bi020417c.**](https://doi.org/10.1021/bi020417c.)​
6. Li H, Jamal J, Plaza C, Pineda SH, Chreifi G, Jing Q, Cinelli MA, Silverman RB, Poulos TL. Structures of human constitutive nitric oxide synthases. Acta Crystallogr, Sect D: Biol Crystallogr. 2014;70:2667–2674. [**https://doi.org/10.1107/S1399004714017064.**](https://doi.org/10.1107/S1399004714017064.)​
7. Do HT, Li H, Chreifi G, Poulos TL, Silverman RB. Optimization of blood brain barrier permeability with potent and selective human neuronal nitric oxide synthase inhibitors having a 2‑aminopyridine scaffold. J Med Chem. 2019;62:2690–2707. [**https://doi.org/10.1021/acs.jmedchem.8b02032.**](https://doi.org/10.1021/acs.jmedchem.8b02032.)​
8. Cheng Y‑C, Prusoff WH. Relationship between the inhibition constant (Ki) and the concentration of inhibitor which causes 50 percent inhibition (IC50) of an enzymatic reaction. Biochem Pharmacol. 1973;22:3099–3108. [**https://doi.org/10.1016/0006-2952(73)90196-2.[7**](https://doi.org/10.1016/0006-2952(73)90196-2.%5B7)]
9. Leber A, Hemmens B, Klösch B, Goessler W, Raber G, Mayer B, Schmidt K. Characterization of recombinant human endothelial nitric‑oxide synthase purified from the yeast Pichia pastoris. J Biol Chem. 1999;274:37658–37664. [**https://doi.org/10.1074/jbc.274.53.37658.**](https://doi.org/10.1074/jbc.274.53.37658.)​
10. Di L, Kerns EH, Fan K, McConnell OJ, Carter GT. High throughput artificial membrane permeability assay for blood−brain barrier. Eur J Med Chem. 2003;38:223–232. [**https://doi.org/10.1016/S0223-5234(03)00012-6.[9**](https://doi.org/10.1016/S0223-5234(03)00012-6.%5B9)]
11. Cahlíková L, Pérez DI, Štěpánková Š, Chlebek J, Šafratová M, Hošt'álková A, Opletal L. In vitro inhibitory effects of 8‑O‑demethylmaritidine and undulatine on acetylcholinesterase and their predicted penetration across the blood−brain barrier. J Nat Prod. 2015;78:1189–1192. [**https://doi.org/10.1021/acs.jnatprod.5b00191.**](https://doi.org/10.1021/acs.jnatprod.5b00191.)​
12. Könczöl Á, Müller J, Földes E, Béni Z, Végh K, Kéry Á, Balogh GT. Applicability of a blood−brain barrier specific artificial membrane permeability assay at the early stage of natural product‑based CNS drug discovery. J Nat Prod. 2013;76:655–663. [**https://doi.org/10.1021/np300882f.**](https://doi.org/10.1021/np300882f.)​
13. Müller J, Esső K, Dargó G, Könczöl Á, Balogh GT. Tuning the predictive capacity of the PAMPA‑BBB model. Eur J Pharm Sci. 2015;79:53–60. [**https://doi.org/10.1016/j.ejps.2015.08.007.**](https://doi.org/10.1016/j.ejps.2015.08.007.)​
14. Friesner RA, Banks JL, Murphy RB, Halgren TA, Klicic JJ, Mainz DT, Repasky MP, Knoll EH, Shelley M, Perry JK. Glide: a new approach for rapid, accurate docking and scoring. 1. Method and assessment of docking accuracy. J Med Chem. 2004;47(7):1739–1749. [**https://doi.org/10.1021/jm0306430.**](https://doi.org/10.1021/jm0306430.)​
15. Friesner RA, Murphy RB, Repasky MP, Frye LL, Greenwood JR, Halgren TA, Sanschagrin PC, Mainz DT. Extra precision glide: docking and scoring incorporating a model of hydrophobic enclosure for protein−ligand complexes. J Med Chem. 2006;49(21):6177–6196. [**https://doi.org/10.1021/jm051256o.**](https://doi.org/10.1021/jm051256o.)​
16. Berman HM, Westbrook J, Feng Z, Gilliland G, Bhat TN, Weissig H, Shindyalov IN, Bourne PE. The protein data bank. Nucleic Acids Res. 2000;28(1):235–242. [**https://doi.org/10.1093/nar/28.1.235.**](https://doi.org/10.1093/nar/28.1.235.)​
17. Madhavi Sastry G, Adzhigirey M, Day T, Annabhimoju R, Sherman W. Protein and ligand preparation: parameters, protocols, and influence on virtual screening enrichments. J Comput Aided Mol Des. 2013;27:221–234. [**https://doi.org/10.1007/s10822-013-9644-8.**](https://doi.org/10.1007/s10822-013-9644-8.)​
18. Jacobson MP, Pincus DL, Rapp CS, Day TJ, Honig B, Shaw DE, Friesner RA. A hierarchical approach to all‐atom protein loop prediction. Proteins. 2004;55(2):351–367. [**https://doi.org/10.1002/prot.10613.**](https://doi.org/10.1002/prot.10613.)​
19. Lu C, Wu C, Ghoreishi D, Chen W, Wang L, Damm W, Ross GA, Dahlgren MK, Russell E, Von Bargen CD. OPLS4: Improving force field accuracy on challenging regimes of chemical space. J Chem Theory Comput. 2021;17(7):4291–4300. [**https://doi.org/10.1021/acs.jctc.1c00302.**](https://doi.org/10.1021/acs.jctc.1c00302.)​
20. Bowers KJ, Chow E, Xu H, Dror RO, Eastwood MP, Gregersen BA, Klepeis JL, Kolossvary I, Moraes MA, Sacerdoti FD. Scalable algorithms for molecular dynamics simulations on commodity clusters. In Proceedings of the 2006 ACM/IEEE Conference on Supercomputing; 2006; pp 84–es. [**https://doi.org/10.1145/1188455.1188544.**](https://doi.org/10.1145/1188455.1188544.)​
21. Martyna GJ, Klein ML, Tuckerman M. Nosé–Hoover chains: The canonical ensemble via continuous dynamics. J Chem Phys. 1992;97(4):2635–2643. [**https://doi.org/10.1063/1.463940.**](https://doi.org/10.1063/1.463940.)​
22. Martyna GJ, Tobias DJ, Klein ML. Constant pressure molecular dynamics algorithms. J Chem Phys. 1994;101(5):4177–4189. [**https://doi.org/10.1063/1.467468.**](https://doi.org/10.1063/1.467468.)​
23. Awasthi A, Patel A, Ha L, Li H, Hardy C, Poulos T, Ansari A, Yang S, Silverman RB. New inhibitors of neuronal nitric oxide synthase for the treatment of melanoma. J Med Chem. 2026. Accepted; [**https://doi.org/10.1021/acs.jmedchem.5c02154.**](https://doi.org/10.1021/acs.jmedchem.5c02154.)​
